# Expression Profiling of MAP Kinase–Mediated Meiotic Progression in Caenorhabditis elegans

**DOI:** 10.1371/journal.pgen.0020174

**Published:** 2006-11-10

**Authors:** Stefanie W Leacock, Valerie Reinke

**Affiliations:** Department of Genetics, Yale University School of Medicine, New Haven, Connecticut, United States of America; Stanford University School of Medicine, United States of America

## Abstract

The LET-60 (Ras)/LIN-45 (Raf)/MPK-1 (MAP kinase) signaling pathway plays a key role in the development of multiple tissues in Caenorhabditis elegans. For the most part, the identities of the downstream genes that act as the ultimate effectors of MPK-1 signaling have remained elusive. A unique allele of *mpk-1, ga111,* displays a reversible, temperature-sensitive, tissue-specific defect in progression through meiotic prophase I. We performed gene expression profiling on *mpk-1(ga111)* animals to identify candidate downstream effectors of MPK-1 signaling in the germ line. This analysis delineated a cohort of genes whose expression requires MPK-1 signaling in germ cells in the pachytene stage of meiosis I. RNA in situ hybridization analysis shows that these genes are expressed in the germ line in an MPK-1-dependent manner and have a spatial expression pattern consistent with the location of activated MPK-1. We found that one MPK-1 signaling-responsive gene encoding a C_2_H_2_ zinc finger protein plays a role in meiotic chromosome segregation downstream of MPK-1. Additionally, discovery of genes responsive to MPK-1 signaling permitted us to order MPK-1 signaling relative to several events occurring in pachytene, including EFL-1/DPL-1 gene regulation and X chromosome reactivation. This study highlights the utility of applying global gene expression methods to investigate genes downstream of commonly used signaling pathways in vivo.

## Synopsis

In many tissues in developing organisms, signaling pathways interpret extracellular cues that change how genes are expressed inside the nucleus and thus direct the appropriate developmental choice. Identification of the genes that are responsive to signaling pathways is critical for understanding how these pathways can promote the correct cell fate. Additionally, understanding the relationships between different regulatory pathways will also help to decipher the network of gene expression that underlies development. The nematode Caenorhabditis elegans has many signaling pathways that are highly similar to those acting in mammals. In particular, the Ras/Raf/MAP kinase signaling pathway acts in many tissues in C. elegans to direct a diverse set of cell fates. Here, we identify a set of genes whose expression alters in response to Ras/Raf/MAP kinase signaling in the germ line during meiosis. We show that this set of genes is primarily expressed in the germ line and that at least one of these genes is important for proper germ cell fate downstream of Ras/Raf/MAP kinase signaling. We also find that the Ras/Raf/MAP kinase signaling pathway functions independently of a second regulatory pathway, the E2F pathway, that acts at a similar time during germ cell development.

## Introduction

Signal transduction pathways play key roles in specifying cell fates. Most signaling pathways terminate in the nucleus and alter the expression of a set of genes that are the ultimate effectors of cellular function. Commonly used signaling pathways have the ability to direct distinct outcomes in diverse tissues, often by regulating tissue-specific programs of gene expression. However, these tissue-specific effectors have been difficult to discover using genetic approaches, perhaps because they are often required for cell viability or are functionally redundant. Microarray analysis provides an excellent approach to identify target genes of signaling pathways because it comprehensively examines the expression of most genes in the genome in parallel without relying on gene function. In particular, application of this technology to the Caenorhabditis elegans germ line provides an excellent opportunity to explore the targets of signaling pathways regulating reproduction.

Conserved regulatory pathways direct the proper temporal and spatial regulation of diverse events in C. elegans germ cell development, including mitosis, meiosis, and gametogenesis. In the distal-most region of the adult germ line, GLP-1(Notch) signaling promotes proliferation [1]. As germ cells progress proximally, they move away from this signal into the transition zone and enter meiotic prophase I. A number of conserved regulatory molecules or pathways function at approximately the same time in the pachytene stage of meiosis I. The E2F-like transcription factor EFL-1 is expressed specifically in pachytene nuclei and, as a heterodimer with its partner DPL-1 (DP), is required for normal fertilization and embryogenesis [2,3]. GLD-1, an RNA-binding protein required for proper meiotic progression and oogenesis, is also present during pachytene and prevents premature translation of mRNAs that encode factors important for oogenesis [4]. GLD-1 is down-regulated in late pachytene, permitting translation of these mRNAs as germ cells develop into oocytes [4]. Two other important events in germ cell development occur in late pachytene. A fraction of presumptive oocytes undergo physiological cell death, mediated by CED-3 and CED-4 [5]. Additionally, the X chromosomes, which have been held transcriptionally silent at earlier stages of germ cell development by the MES proteins, become globally competent for transcription [6,7]. The factors that promote X chromosome chromatin remodeling during late pachytene are unknown.

Of particular importance for this work, the Ras/MAP kinase signaling pathway also functions during pachytene to promote meiotic progression. Mutation of any of the core genes in the MAP kinase signaling pathway—*let-60* (Ras), *lin-45* (Raf), *mek-2* (MEK), or *mpk-1* (MAP kinase)—results in failure of germ cells to progress from pachytene into oogenesis [8–10]. Studies in other systems have shown that activated MAP kinase can phosphorylate either cytoplasmic substrates such as ribosomal S6 kinase, or nuclear transcription factors, resulting in the activation or repression of key target genes (e.g., [11,12]). The phosphorylation substrates of MPK-1 in the C. elegans germ line, as well as other downstream effectors that are required for meiotic progression, are still unknown. Activated MPK-1 does translocate to pachytene nuclei and thus at least some substrates are likely to be nuclear proteins [2,13]. Additionally, germ cells in pachytene are transcriptionally active [14] and are therefore competent to have a transcriptional response to MPK-1 signaling. After MPK-1 signaling occurs, germ cells exit pachytene, progress through diplotene, enter diakinesis, and mature into oocytes, which are transcriptionally silent. Thus, any genes regulated in response to MPK-1 signaling are expressed during a relatively brief window of development.

In this study, we use DNA microarray analysis to identify a set of MPK-1 signaling-responsive genes that have reduced expression in the absence of MAP kinase signaling and increased expression as MAP kinase signaling resumes. Independent verification by in situ analysis demonstrates that many of these genes have expression in the germ line that is first detectable in the pachytene stage and is MPK-1 signaling-dependent. We examined the function of three of these MPK-1 signaling-responsive genes and found that mutation of a gene we have designated as *paired zinc finger-1 (pzf-1)* enhances the phenotype of an *mpk-1* mutant, indicating that we successfully identified genes that function with *mpk-1* in the germ line. Comparison of MPK-1 signaling-responsive genes with EFL-1/DPL-1 target genes reveals that these two pathways generally regulate expression of distinct sets of genes. Finally, we use our results to examine the temporal relationship between MPK-1 signaling and other events in the pachytene stage of meiosis I. We find that EFL-1/DPL-1 regulation of gene expression occurs prior to MPK-1 activation. MPK-1 signaling in turn must occur for expression of many genes on the X chromosome, as well as for translation of the oogenesis protein RME-2. These results further refine our understanding of the relationships between conserved regulatory pathways and developmental events in the germ line.

## Results

### The *mpk-1(ga111)* Pachytene Exit Phenotype Is Temperature-Sensitive and Reversible

**Figure 1:**
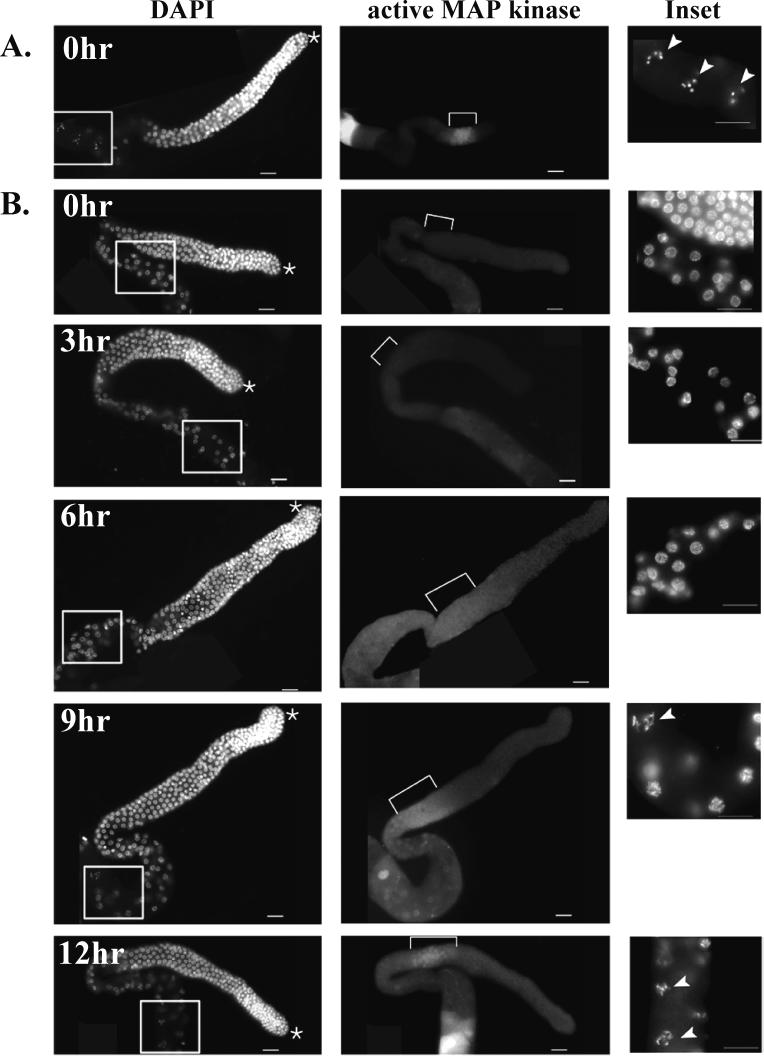
Meiotic Progression and MAP Kinase Activation Is Restored after Shift to Permissive Temperature Dissected gonads were stained with DAPI (left column) and α-diP MAP kinase (middle column). Boxed area in left column is enlarged in right column (inset) to show germ cell morphology during meiotic progression. Asterisks indicate distal end of germ line. Arrowheads indicate nuclei in diakinesis. Brackets indicate region with activated MAP kinase staining. (A) Gonads from control animals. (B) *mpk-1* gonads at 26 °C (0 h) and following shift to 15 °C (3–12 h). All gonads also carry *fem-1(hc17)* and *unc-79(e1068)* alleles. Scale bar, 20 μm.

To investigate events occurring as germ cells progress from the pachytene to diakinesis stage of meiosis I, we took advantage of a temperature-sensitive, loss-of-function allele of MAP kinase, *mpk-1(ga111),* that produces a phenotype only in the germ line [9]. When grown at 15 °C or 20 °C, *mpk-1(ga111)* mutants are fertile with reduced brood size (unpublished data). The same mutants raised at the restrictive temperature of 26 °C are completely sterile and exhibit a pachytene exit defect (Pex) phenotype (Figure 1) [8]. They produce variable numbers of sperm but no oocytes because germ cells fail to proceed beyond the pachytene stage of meiosis I in adults. In the experiments described below, additional mutations are also included. First, the *mpk-1(ga111)* allele is linked to a mutation in *unc-79* (for simplification, this double mutant is hereafter termed “*mpk-1*”) [9]. Second, we included the *fem-1(hc17)* mutation, which blocks spermatogenesis at 26 °C, to avoid the variability in sperm number and to focus on events occurring during oogenesis [15].

**Figure 2:**
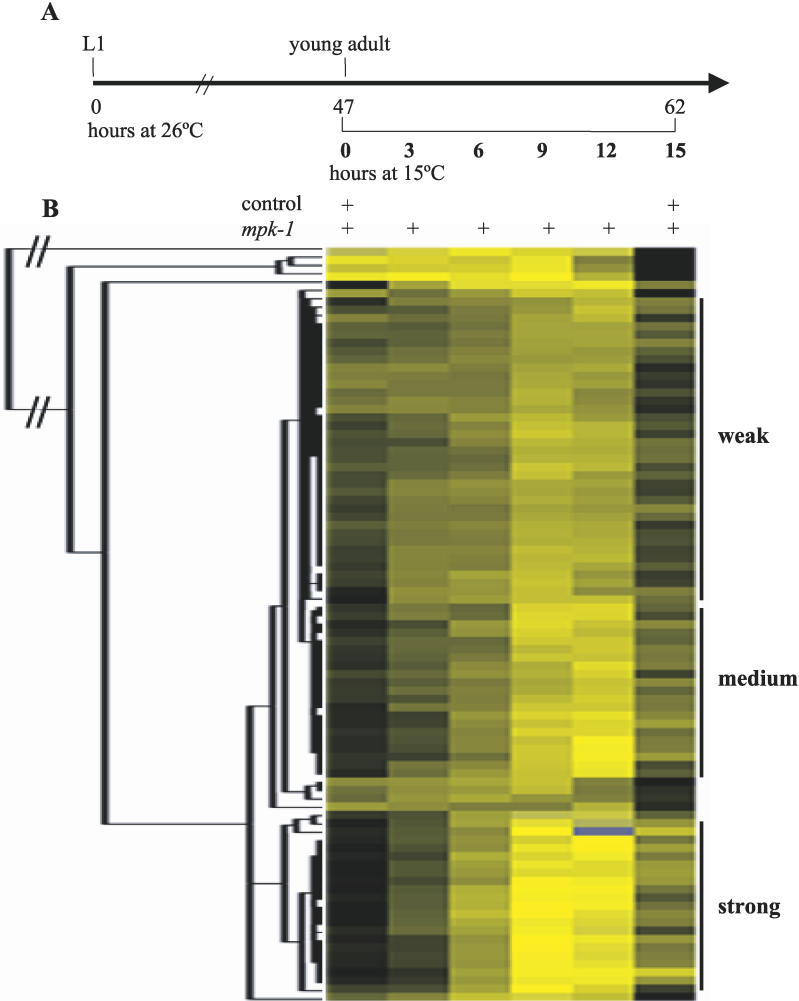
Temporal Expression Profiling of Meiotic Progression (A) Adults were shifted to permissive temperature (47 h after hatching) and subsequently analyzed at indicated times. (B) Hierarchical clustering analysis of expression profiles upon resumption of MPK-1 signaling. Each column represents the mean of three replicates for each time sampled. Each row represents one of 90 genes with enriched expression in control compared with *mpk-1* at 0 h. Low expression in *mpk-1* relative to the control (black) is evident at 0 h, with increasing expression as meiotic progression resumes (yellow). Three major clusters, each with an expression correlation > 0.95, are indicated (strong, medium, and weak).

A critical feature of *mpk-1(ga111)* is the reversibility of the Pex phenotype. We found that when *mpk-1* animals grown to adulthood at the restrictive temperature (26 °C) are subsequently incubated at the permissive temperature (15 °C), meiotic progression can resume (see Figure 2A for diagram of timeline). Using DAPI staining to observe the morphology of germ nuclei, we examined *mpk-1* animals at 3-h intervals after transfer to 15 °C. *mpk-1* gonads are Pex prior to incubation at the permissive temperature (0 h) and remain Pex even after 6 h (Figure 1; Table 1). After 9 h at 15 °C, we observed increasing chromosome condensation suggestive of the diplotene stage of meiosis I. After 12 h, chromosomes appeared to be in diplotene or diakinesis in most gonads (Figure 1), and by 15 h all *mpk-1* gonads had enlarged germ cells characteristic of oocytes present in the proximal arm. These oocytes could be fertilized and generated viable offspring when provided with sperm from wild-type males.

**Table 1:**
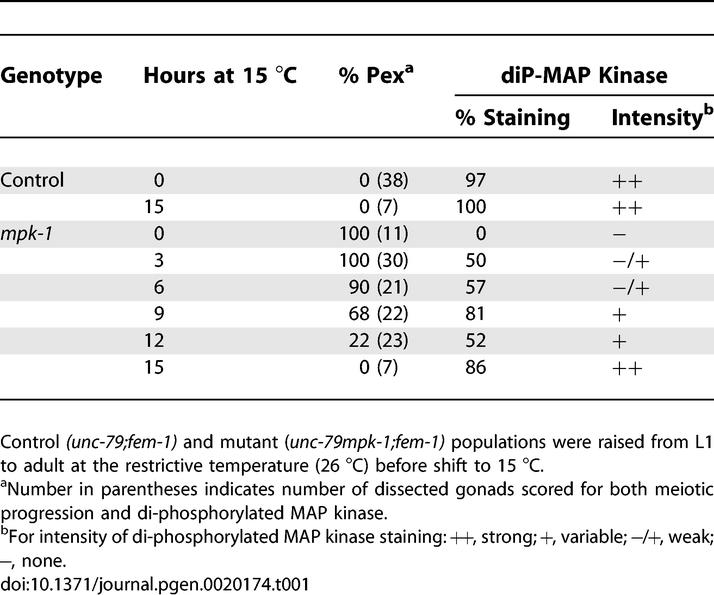
Quantification of Meiotic Progression and MAP Kinase Activation after Shift of *mpk-1* to Permissive Temperature

To correlate the onset of active MAP kinase with meiotic progression, we used an antibody specific for the activated, di-phosphorylated form of MAP kinase [16] (Figure 1; Table 1). This antibody stains two regions of wild-type gonads where MPK-1 is active, pachytene germ cells and maturing oocytes [2,13]. No staining was present in Pex gonads of *mpk-1* adults raised at 26 °C. Three hours after these animals were shifted to the permissive temperature, very weak, diffuse staining was detected. Signal strength and frequency increased at 6 and 9 h, and after 12 h, activated MAP kinase staining was as strong as in control animals. Thus, detection of active MAP kinase at the pachytene region of the germ line preceded the resumption of meiotic progression as determined by nuclear and cellular morphology. We concluded that *mpk-1* mutants could be used to control the onset of meiotic progression, allowing us to investigate events occurring after exit from the pachytene stage in the germ line.

### Identification of MPK-1 Signaling-Responsive Genes

To define some of the key events underlying the transition of germ cells from the pachytene stage into oogenesis, we used whole-genome DNA microarrays to investigate gene expression profiles in *mpk-1* mutants [17]. Each hybridization compared a control *(unc-79;fem-1)* or *mpk-1 (unc-79mpk-1;fem-1)* sample to a common reference RNA sample. Thus, all comparisons between control and *mpk-1* samples discussed below are indirect, using the reference as a common denominator (see Materials and Methods). Triplicate hybridizations were performed for each comparison from independently grown and isolated control and *mpk-1* populations.

We first compared control and *mpk-1* samples raised at the restrictive temperature (Pex; 0 h in Figure 2A) and identified a core set of 90 genes with enriched expression in the control population relative to *mpk-1* (>1.6-fold, Student's *t*-test *p* < 0.05; Table S1). These genes likely require MPK-1 signaling and/or meiotic progression for normal expression levels. Strikingly, only seven genes had higher expression in *mpk-1* mutants relative to controls, suggesting that few genes are down-regulated as germ cells exit the pachytene stage of meiosis I.

Genes most likely to be relevant to meiotic progression and oogenesis should be sensitive to the restoration of *mpk-1* activity in the germ line. To investigate how the expression of these 90 genes responds as meiotic progression resumes, we collected RNA samples from *mpk-1* young adults at 3-h intervals (3, 6, 9, 12, and 15 h) following the shift to permissive temperature (Figure 2A). To rule out the possibility that any gene expression changes occur independently of meiotic progression, we compared the control strain at 0 h (restrictive temperature) to the control strain at 15 h (permissive temperature). None of the 90 genes had significant expression changes in this control comparison, indicating that their expression was not affected by time or temperature differences in our experiment.

We then grouped these 90 genes by similarity in expression profile over the 15-h span using hierarchical clustering [18]. In total, 79/90 genes had increased expression within 12 h after restoration of meiotic progression. These 79 genes can be divided into three classes that we have termed strong, medium, and weak responders (Figure 2B). Strong responders (21 genes) showed increased expression within 6 h following the shift to permissive temperature, with an even more dramatic increase in expression at later times. Notably, increased expression occurred prior to any visible alteration in the Pex phenotype, suggesting that these changes were an early event in response to MPK-1 activation. The 21 medium responders displayed moderately increased expression levels by 9 h after the shift to 15 °C, while the 37 weak responders showed slightly smaller, later changes in expression levels (Figure 2B). The remaining 11 genes that showed essentially no change over the course of the experiment were considered nonresponsive to the restoration of MPK-1 activity. We refer to the set of 79 responsive genes as “MPK-1 signaling-dependent” or “MPK-1 signaling-responsive.” MPK-1 could play a relatively direct role in regulating their expression by phosphorylating transcription factors that act on these genes. Alternatively, their regulation could be an indirect or downstream consequence of meiotic progression that occurs subsequent to MPK-1 signaling. However, the amount of time to elicit indirect effects is limited by the brief window between MPK-1 activation in late pachytene and transcriptional silencing of all chromosomes in diakinesis.

### Germ Line Expression of MPK-1 Signaling-Responsive Genes

We next asked whether MPK-1 signaling-responsive genes are normally expressed in the germ line by examining existing microarray data that measured expression in the oogenic germ line relative to the soma [19]. We also examined whole-mount in situ hybridization images that are available for many of the genes in an online database (http://nematode.lab.nig.ac.jp). We found that 65% of the MPK-1 signaling-responsive genes had oogenic germ-line-enriched expression, a ~4-fold over-representation from the expected number (*p* < 0.001; Table S1). In particular, 95% of strong responders had oogenic germ-line-enriched expression, compared to 61% of medium and 54% of weak responders. Of the MPK-1 signaling-responsive genes that did not display oogenic germ-line-enriched expression by microarray analysis, 16 had available whole-mount in situ hybridization images; of these, nine exhibited germ line staining (Table S1). These two lines of evidence together indicate that 84% of MPK-1 signaling-responsive genes are expressed in the germ line. By contrast, nine of the 11 nonresponsive genes did not have germ line expression by either in situ or microarray analysis. These genes could be false positives or genes that require MPK-1 signaling in somatic tissues.

Additionally, we compared the MPK-1 signaling-responsive genes to a set of 708 C. elegans genes identified in a previous microarray experiment measuring gene regulation upon expression of activated LET-60 (Ras), and hence MPK-1, during early larval development [20]. We found an overlap of only one gene—*sea-1/tbx-18*. This limited overlap suggests that Ras/MPK-1 signaling has very specific gene targets in different tissues at different times in development.

### MPK-1 Signaling Is Required for Expression of Candidate Genes on Autosomes

To verify through an independent approach that the candidate genes are responsive to MPK-1 signaling and meiotic progression, we examined in detail their spatial expression pattern using in situ hybridization. For this analysis, we focused only on genes on autosomes, because the X chromosomes undergo global chromatin remodeling that complicates the interpretation of X-linked gene expression patterns (see below). The expression of MPK-1 signaling-dependent genes should initiate downstream of the domain of activated MAP kinase in the pachytene region of the germ line. Consistent with this expectation, we found that 11 of the 30 genes with visible expression patterns in the database (http://nematode.lab.nig.ac.jp) displayed expression that was first detectable in the late pachytene region near the bend in the gonad arm, coincident with the approximate location of MPK-1 activity, and persisting throughout oogenesis. Previous examination of 20 randomly selected autosomal oogenic germ-line-enriched genes had not identified any with proximal gonad-restricted expression [6], suggesting that MPK-1 signaling-responsive genes are enriched for this rare expression pattern. This expression pattern was particularly prominent among autosomal genes in the strong and medium responder groups: 10/12 showed proximal-restricted expression, whereas only 1/16 weak responders had proximal-restricted expression.

**Figure 3:**
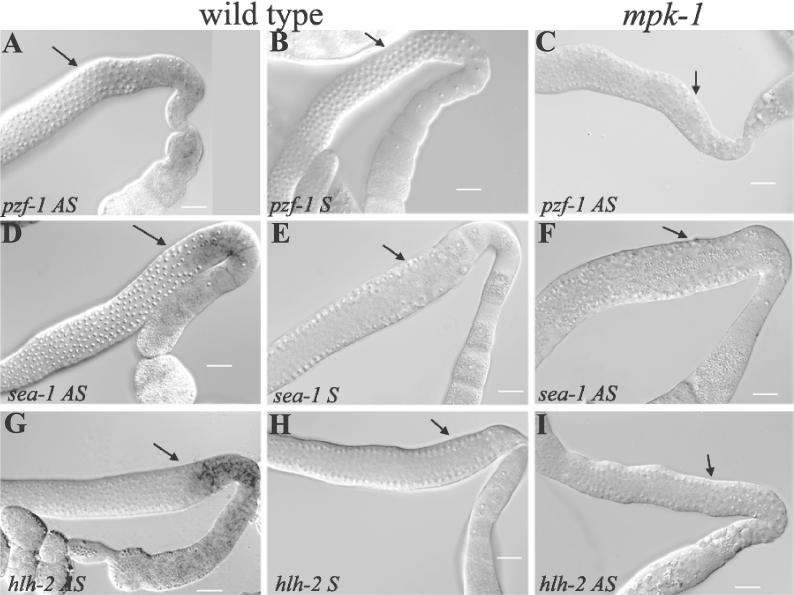
Candidate MPK-1 Signaling-Responsive Autosomal Genes Depend on *mpk-1* for Expression RNA in situ hybridizations of N2 (A, B, D, E, G, and H) and *mpk-1* (C, F, and I) gonads. Sense and AS probes correspond to three MPK-1 signaling-responsive genes on autosomes: *pzf-1* (A–C), *sea-1/tbx-18* (D–F), and *hlh-2* (G–I). Only medial and proximal arm of gonads are shown; no staining was detected in the distal gonad for any probe. Arrow indicates location of late pachytene region. Scale bar, 20 μm.

We directly tested whether representatives of the strong, medium, and weak responder groups were indeed responsive to MPK-1 signaling by performing in situ hybridization in wild-type and *mpk-1* dissected gonads. Three genes—*pzf-1, sea-1/tbx-18,* and *hlh-2* (a strong, medium, and weak responder, respectively)—did not have staining patterns available in the online database. In wild-type gonads, we detected strong and consistent expression using antisense (AS) probes for all three genes, while sense probes did not produce any signal (Figure 3). Detectable expression of all three genes was limited to the bend and proximal region of the gonad, corresponding with the approximate region of MPK-1 activation. In gonads from *mpk-1* animals raised at the restrictive temperature, expression of all three candidates was largely decreased or absent (Figure 3; Table 2). Thus, all three genes require MPK-1 signaling for normal levels of expression. Because we tested relatively few genes, we cannot reliably calculate the percentage of MPK-1-responsive genes from the candidate group as a whole. However, these genes were not chosen based on any expectation or prior knowledge of their expression. The fact that we observed MPK-1 signaling-dependent expression for all three randomly chosen genes suggests that the expression of many untested candidate autosomal genes likely also depends upon MPK-1 signaling. To control for the possibility that any gene expressed during oogenesis is nonspecifically decreased in *mpk-1,* we examined the expression of *rme-2,* which encodes the yolk receptor [21]. *rme-2* is normally expressed in the medial and proximal germ line [4] but was not differentially expressed in the *mpk-1* microarray experiments. In situ hybridization of *rme-2* showed similar expression in both wild-type and *mpk-1* gonads.

**Table 2:**
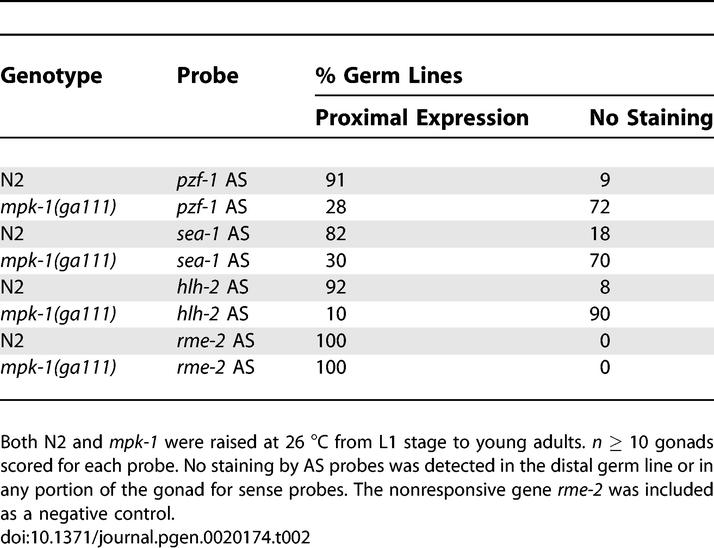
In Situ Hybridization Analysis of Autosomal MPK-1 Signaling-Responsive Genes

### Categorization of MPK-1 Signaling-Responsive Gene Function

We examined the predicted functions of the proteins encoded by the 79 MPK-1 signaling-responsive genes. With the exception of genes with unknown function, the most prominent functional groups are those encoding factors involved in proteolysis (16 genes) and DNA/RNA regulation (14 genes). The majority of genes encoding DNA/RNA regulatory proteins are strong or medium responders (64%), whereas those encoding proteolysis factors tend to be weak responders (82%) (Table S1). We also examined RNA interference phenotypes of the 79 candidates based on published large-scale RNA interference screens [22–26]. Nineteen genes displayed a phenotype in at least one screen, with the most common phenotype, partially penetrant embryonic lethality, occurring for 13 genes. This observation suggests that many candidate genes are expressed in the proximal germ line in order to load their gene products into the oocyte for function in the early embryo.

### Mutation of the Strong Responder *pzf-1* Enhances the *mpk-1* Brood Size Defect

The predicted functions and RNA interference phenotypes of the MPK-1 signaling-responsive genes did not identify clear candidates likely to function in meiotic progression. Because of the prevalence of DNA/RNA regulatory proteins encoded by MPK-1 signaling-responsive genes, we decided to investigate the potential involvement of three such candidates in meiotic progression. We isolated deletion mutants in three genes encoding predicted DNA/RNA regulatory proteins that displayed proximal-restricted expression by in situ hybridization. We found that mutation of the strong responder T05G11.1 displayed a phenotype in our assays, whereas mutation of *sea-1/tbx-18* and T08D10.2, two medium responders, did not.

T05G11.1 is an autosomal gene that is predicted to encode a protein with three pairs of C_2_H_2_ zinc fingers. This organization categorizes it as a member of the “separated-paired” class of C_2_H_2_ zinc finger proteins, which includes transcription factors such as *Drosophila* Tramtrack and mammalian basonuclin [27]. Based on this primary distinguishing feature of T05G11.1, we have designated this gene *paired zinc finger-1 (pzf-1).*

**Figure 4:**
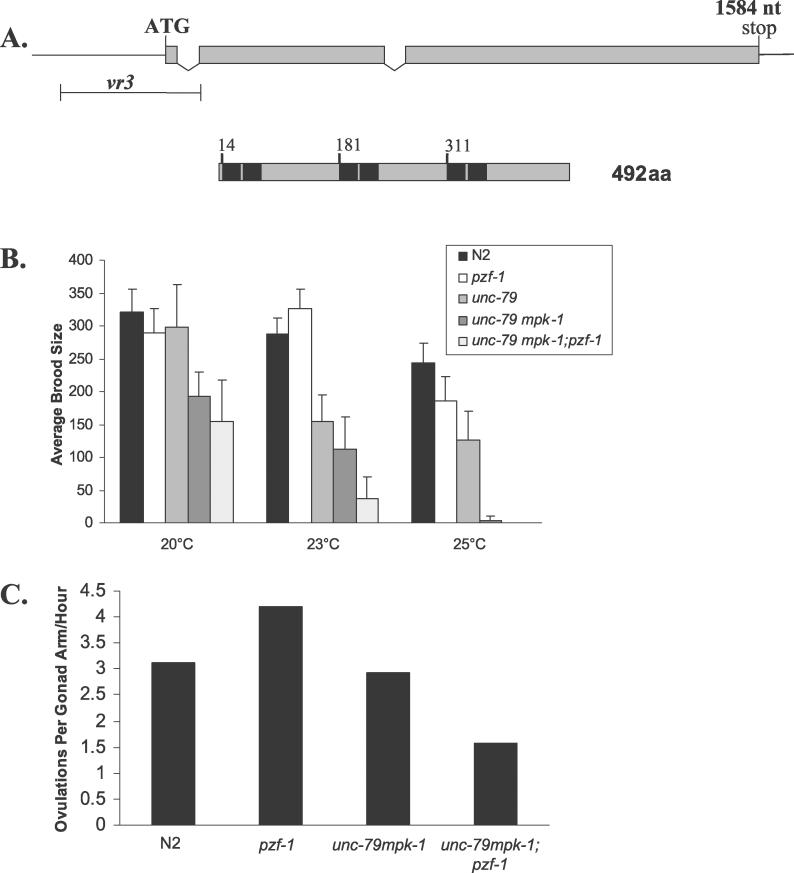
*pzf-1* Displays a Reduced Brood Size in the *mpk-1* Background (A) Schematic of *pzf-1* genomic locus showing location of *vr3* deletion (top) and predicted PZF-1 protein (bottom), with predicted C_2_H_2_ zinc finger domains in black. Numbers indicate amino acid residue of start of each C_2_H_2_ zinc finger pair. (B) Chart of mean live progeny per animal of N2, *pzf-1(vr3), unc-79(e1068)*, *unc-79(e1068)mpk-1(ga111),* and *unc-79(e1068)mpk-1(ga111);pzf-1(vr3)* at 20 °C, 23 °C, and 25 °C. The total number of progeny from six to 24 hermaphrodites was counted for each temperature. Error bars indicate standard deviation. (C) Ovulations/gonad arm/hour of N2, *pzf-1(vr3), unc-79(e1068)mpk-1(ga111),* and *unc-79(e1068)mpk-1(ga111);pzf-1(vr3).* The *unc-79(e1068)mpk-1(ga111)* strain showed a statistically significant difference from *unc-79(e1068)mpk-1(ga111);pzf-1(vr3)*. Because ovulation rates were determined on groups of N2 and *pzf-1(vr3)* worms, standard deviation was not calculated.

Using PCR screening of a deletion library, we isolated a mutant allele of *pzf-1, vr3,* that removes 309 nucleotides upstream of the start of translation, as well as the first 88 nucleotides of the *pzf-1* coding sequence (Figure 4A). Thus, *pzf-1(vr3)* presumably lacks key core promoter elements and is not expressed. In agreement with this expectation, microarray analysis of *pzf-1* mutant adults demonstrated that the *pzf-1* transcript was reduced to levels close to background in the *pzf-1* mutant relative to wild-type (unpublished data).

*pzf-1(vr3)* mutants are superficially wild-type and have a normal brood size (Figure 4B). If *pzf-1* acts downstream of *mpk-1,* we reasoned that loss of *pzf-1* activity might enhance a weak *mpk-1* phenotype. We built a double mutant between *mpk-1(ga111)* and *pzf-1(vr3)* and examined brood size. Because *mpk-1* animals are essentially sterile at 25 °C, we examined both *mpk-1* and *mpk-1;pzf-1* adults at temperatures at which *mpk-1* was impaired but retained some activity. At the relatively permissive temperature of 20 °C, *mpk-1* had a reduced live brood size relative to control (192 versus 299 progeny), which was further decreased in *mpk-1;pzf-1* double mutants to 153 (Figure 4B). At an intermediate temperature of 23 °C, the brood size of *mpk-1* was even more dramatically reduced in the absence of *pzf-1* (from 112 to 37; *p* < 0.0001). The reduction in brood size by *pzf-1* is specific, because we did not see reduction when we combined *mpk-1* with the *sea-1/tbx-18(vr4)* allele (unpublished data). However, *pzf-1(vr3)* did not suppress a gain-of-function mutation in *let-60* (Ras) or a loss-of-function mutation in the MPK-1 inhibitor *lip-1(zh15),* both of which result in elevated levels of MPK-1 activity [28,29] (unpublished data). We conclude that the role of *pzf-1* is revealed when MPK-1 activity is partially impaired, but that loss of *pzf-1* is not sufficient to overcome the effects of elevated MPK-1 activity in the Ras gain-of-function or *lip-1* mutants.

We investigated possible reasons for the decreased brood size of the *mpk-1;pzf-1* double mutant. First we examined the incidence of apoptosis in germ cells by monitoring incorporation of SYTO12 dye [5] and found that the *mpk-1;pzf-1* mutant germ line displayed levels equivalent to controls (unpublished data). We then measured the rate of progeny production in *mpk-1* and *mpk-1;pzf-1* mutants at 23 °C, and found that loss of *pzf-1* caused a significant decrease (from 2.9 to 1.6 progeny/hour, *p* < 0.05; Figure 4C). This decrease could be caused by a reduction either in the rate at which oocytes are produced (meiotic progression) or in the rate of oocyte maturation and/or ovulation. If the ovulation rate but not meiotic progression is reduced, then unovulated oocytes could accumulate in the proximal gonad [30]. We did not observe excess unovulated oocytes in the proximal gonad of *mpk-1;pzf-1* double mutants, suggesting that ovulation can occur when oocytes are made. These observations suggest that the rate of meiotic progression, and consequently oocyte maturation/ovulation as well, is reduced in the double mutant compared to either single mutant.

We also found that the *mpk-1;pzf-1* double mutant displayed meiotic chromosome nondisjunction at 23 °C. Nondisjunction of any autosome results in embryonic lethality, whereas nondisjunction of the X chromosome results in an increased incidence of XO males [31]. The *mpk-1;pzf-1* double mutants display increased embryonic lethality at 23 °C relative to *mpk-1* or *pzf-1* single mutants (11% compared to 2% and <1%, respectively; Table 3). Additionally, they also display an increased incidence of males. While males are present at ≤1% in wild-type, *mpk-1,* or *pzf-1* single mutants, we observed 11% male progeny in the *mpk-1;pzf-1* double mutant (Table 3). To determine whether defects in chromosome segregation were occurring, we monitored chromosome number in oocytes. During diakinesis in wild-type oocytes, chromosome homologs are held together as bivalents by the chiasmata that form during recombination, making six bivalent pairs. In *mpk-1;pzf-1* mutants, nuclei in diakinesis displayed an increased incidence of extra chromosomes relative to *mpk-1,* indicating the presence of univalent chromosomes (Table 3). Thus, *pzf-1* likely contributes to, but is dispensable for, proper chromosome segregation during meiosis. Combined, these experiments strongly indicate that *pzf-1* acts downstream of or in parallel to *mpk-1* in multiple events in the germ line. Given that *pzf-1* expression is dependent on MPK-1 signaling, the simplest explanation is that *pzf-1* acts downstream of *mpk-1*.

**Table 3:**
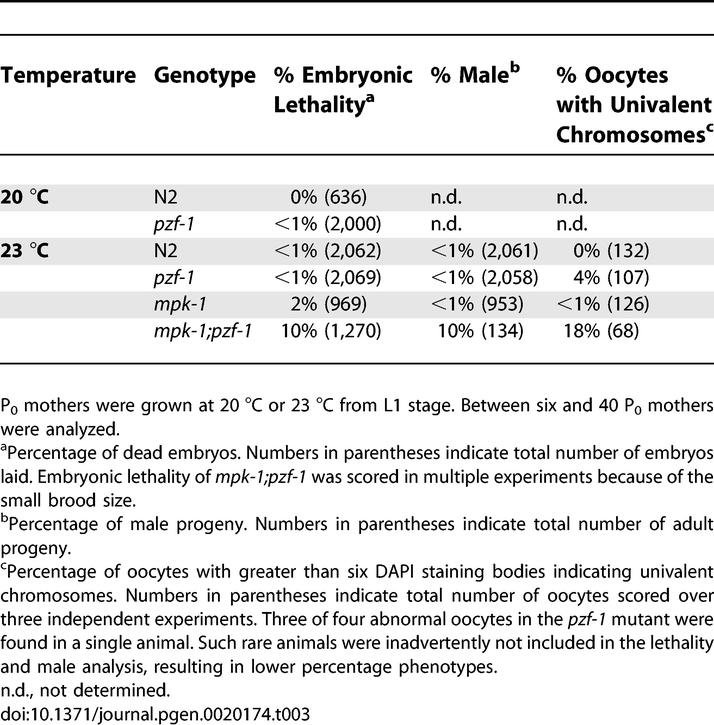
Meiotic Segregation Defects in *mpk-1;pzf-1* Mutants

### X Chromosome Reactivation Occurs Subsequent to MPK-1-Mediated Pachytene Exit

We observed a high frequency of X linkage among MPK-1 signaling-responsive genes, particularly among those with oogenic germ-line-enriched expression [19]. Fully 35% (19/53) are X linked, considerably higher than expected if randomly distributed over all six chromosomes. This bias is even more remarkable because the X chromosomes harbor relatively few genes with oogenic germ-line-enriched expression [19]. Only ~6% of genes with oogenic germ-line-enriched expression are X linked, likely because many genes on the X chromosomes are silenced in the distal and medial germ line and only become active in the proximal germ line [6]. Thus, MPK-1 signaling-responsive genes are located on the X chromosome about 6-fold more frequently than expected.

**Figure 5:**
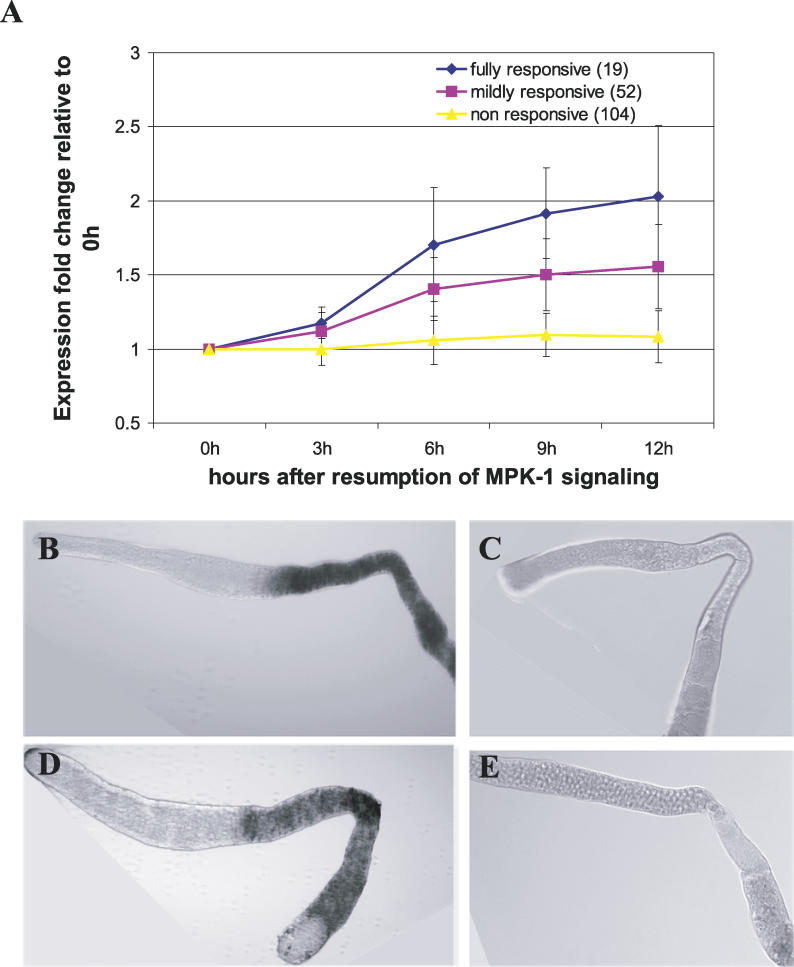
X-Linked Genes Depend on MPK-1 for Germ Line Expression (A) Graph of mean changes in gene expression for X-linked genes in response to MPK-1 signaling. Blue indicates 19 MPK-1 signaling-responsive genes; pink indicates 52 additional X-linked genes with a mild response; and yellow indicates 104 X-linked genes with no response. Error bars indicate standard deviation. (B–E) In situ hybridization of N2 (A and C) and *mpk-1* (B and D) gonads with probes for an X-linked MPK-1 signaling-responsive gene, F35C8.7 (A and B), and an X-linked non-responsive gene, C04A11.3 (C and D). Sense probes showed no signal.

The high frequency of X linkage, coupled with the fact that both MPK-1 activation and the onset of autosomal MPK-1 signaling-responsive gene expression coincide with the onset of expression of many X-linked genes in the proximal germ line, suggested to us that MPK-1 signaling might be required for X chromosome reactivation. However, of 175 X-linked genes with oogenic germ-line-enriched expression [19], only 19 were detected as MPK-1 signaling-responsive in our microarray experiments. Some X-linked genes with a moderate response to MPK-1 signaling could have been missed because they had low expression or because somatic expression masked regulation by MPK-1 signaling in the germ line. We therefore examined more closely the expression profiles of the remaining 156 X-linked genes that did not meet our initial criteria for MPK-1 signaling-dependent expression. We found that another 52 displayed a consistent rise in expression in response to the resumption of MPK-1 signaling, albeit with slightly reduced amplitude compared to the 19 fully responsive genes (mildly responsive, Figure 5A; see Materials and Methods). The remaining 104 X-linked genes had even lower or no response to MPK-1 signaling. Moreover, based on available whole-mount in situ hybridization data (http://nematode.lab.nig.ac.jp), 70% of the fully responsive and 64% of the mildly responsive X-linked genes had proximal-specific germ line expression, compared to 33% of nonresponsive X-linked genes (Table S1). These data suggest that the majority of X-linked genes with proximal germ line expression respond to MPK-1 signaling.

To ensure that X-linked genes truly were responsive to MPK-1 signaling in the germ line, we used in situ hybridization of dissected gonads to examine the expression of eight X-linked genes (representing fully responsive, mildly responsive, and nonresponsive genes) in wild-type and *mpk-1* backgrounds. All eight showed proximal-specific expression in wild-type gonads, and six had a decreased frequency of staining in *mpk-1* gonads at the restrictive temperature (Figure 5B–5E; Table 4). Overall, both the whole-animal microarray and dissected gonad in situ data are consistent with the concept that MPK-1 signaling occurs prior to expression of many X-linked genes. MPK-1 could either play a direct role in global X reactivation or be indirectly required for progression of germ cells through meiosis to the point where X reactivation occurs.

**Table 4:**
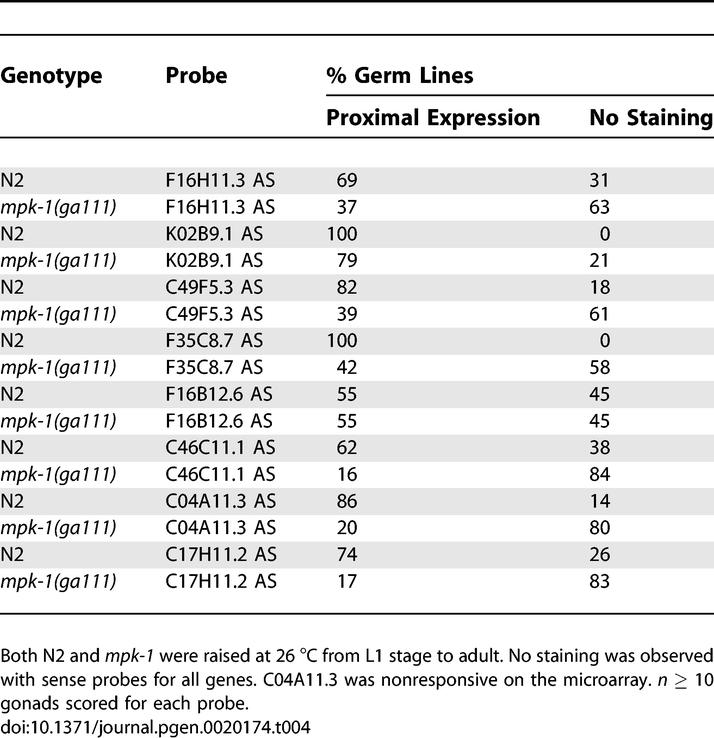
In Situ Hybridization Analysis of X-Linked MPK-1 Signaling-Responsive and Nonresponsive Genes

### MPK-1 Signaling Occurs after EFL-1/DPL-1-Mediated Gene Activation and before GLD-1 Down-Regulation

Another event occurring in the pachytene stage of meiosis I is the regulation of gene expression by the heterodimeric transcription factor EFL-1/DPL-1 (E2F) [2,3]. We wished to determine whether EFL-1/DPL-1 acts before or after MPK-1 signaling. Unlike *mpk-1* mutants, *efl-1* and *dpl-1* mutant germ lines do undergo meiotic progression, only revealing defects at a later stage of oocyte development [3]. Loss-of-function *efl-1* mutants exhibit prolonged MAP kinase activity in oocytes, indicating that EFL-1/DPL-1 is required to attenuate MPK-1 signaling [2]. These observations suggest that EFL-1/DPL-1 acts after MPK-1. Recent expression profiling of *efl-1* and *dpl-1* mutant gonads identified a set of EFL-1/DPL-1-responsive genes, 75 of which are down-regulated in the mutants relative to wild-type and 35 of which are up-regulated [3]. Several of these targets function in late oogenesis. If EFL-1/DPL-1 acts after MPK-1 signaling, genes down-regulated in *efl-1* and *dpl-1* mutants should also have decreased expression in *mpk-1* mutants, because germ cells in *mpk-1* mutants do not proceed through pachytene to a point at which EFL-1/DPL-1 would act. Alternatively, if EFL-1/DPL-1 acts prior to or concomitant with MPK-1 signaling, EFL-1/DPL-1 targets would still be expressed in the *mpk-1* mutant.

**Figure 6:**
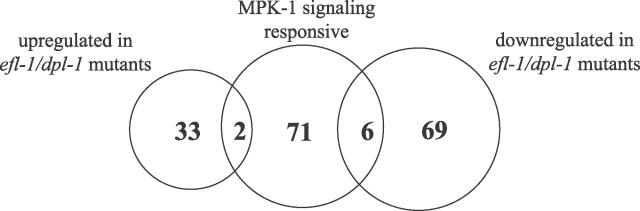
MPK-1 and EFL-1/DPL-1 Do Not Regulate the Same Set of Genes in the Germ Line MPK-1 signaling-responsive genes identified in this study have minimal overlap with candidate down-regulated or up-regulated EFL-1/DPL-1-responsive genes (Table S1).

Comparison of MPK-1 signaling-responsive and EFL-1/DPL-1-responsive genes identified limited overlap (Figure 6). Only eight of the 79 MPK-1 signaling-responsive targets were also EFL-1/DPL-1-responsive; of these, six were down-regulated in *efl-1* and *dpl-1* mutants (Table S1). The fact that most EFL-1/DPL-1-responsive genes are still expressed in *mpk-1* mutants at levels comparable to wild-type indicates that EFL-1/DPL-1 might act at the same time or prior to MPK-1 signaling. We surveyed available in situ hybridization images for autosomal EFL-1/DPL-1-responsive genes and compared them to images of MPK-1 signaling-responsive genes (http://nematode.lab.nig.ac.jp). Thirty of 34 EFL-1/DPL-1-responsive genes had initial expression more distally than the MPK-1 signaling-responsive genes. EFL-1/DPL-1-responsive gene expression begins in the early- to mid-pachytene range [3], while MPK-1 signaling-responsive gene expression is first detectable in late pachytene (this work).

**Figure 7:**
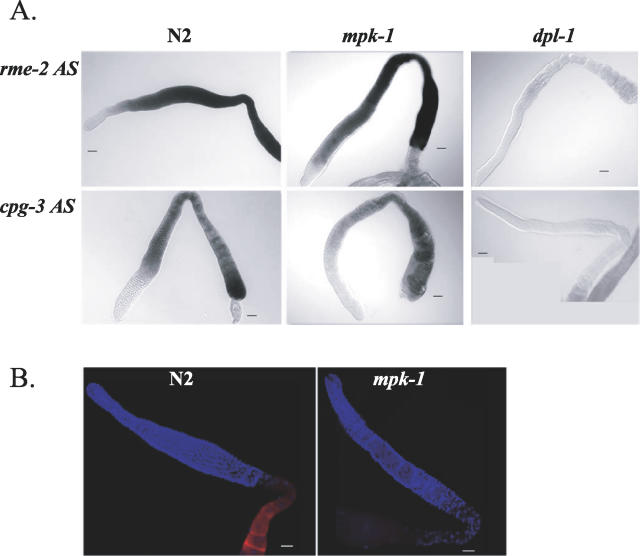
Expression of E2F-Regulated Genes Does Not Require MPK-1 Signaling (A) In situ hybridization of N2, *mpk-1,* and *dpl-1* gonads stained with *rme-2* and *cpg-3* AS probes. Sense probes showed no signal. (B) N2 and *mpk-1* gonads stained with an antibody to the RME-2 protein (red) and DAPI to mark nuclei (blue). Scale bar, 20 μm.

To directly test whether EFL-1/DPL-1-responsive genes were still expressed in an *mpk-1* mutant, we examined two EFL-1/DPL-1 targets, *rme-2* and *cpg-3,* which did not have altered expression in the *mpk-1* microarray analysis. In particular, *rme-2* is one of the most strongly regulated and thoroughly validated EFL-1/DPL-1-responsive genes [3]. Using in situ hybridization, we verified that *rme-2* and *cpg-3* are expressed in *mpk-1* mutants similarly to in wild-type, even though they are undetectable in *dpl-1* mutants (Figure 7A). As with many of the EFL-1/DPL-1-responsive genes that display more distal expression based on the whole-mount in situ analysis, expression of *rme-2* and *cpg-3* is first detectable in the medial germ line, more distal than most MPK-1 signaling-responsive genes (e.g., compare Figure 7A to 3A and 5A; see also [4]).

**Figure 8:**
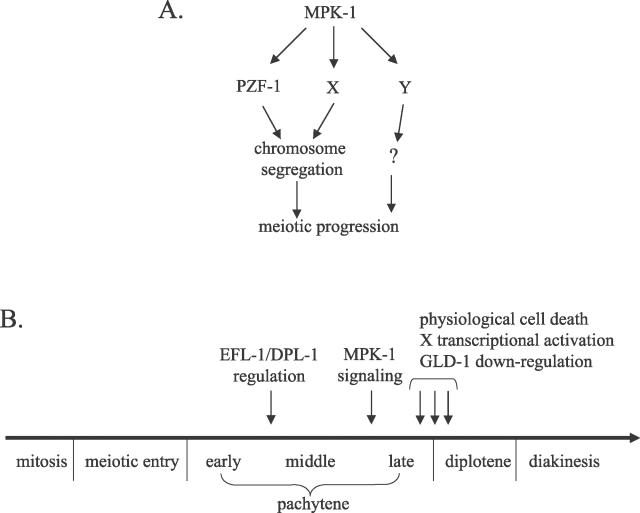
MPK-1 Signaling in the Germ Line (A) Model of multiple MPK-1 signaling-dependent effectors acting together to promote meiotic progression. X and Y represent putative proteins that function downstream of MPK-1. Additional processes besides chromosome segregation that contribute to meiotic progression are indicated by the question mark. (B) Representation of events in germ line development. MPK-1 signaling occurs downstream of EFL-1/DPL-1 gene regulation, but prior to physiological germ cell death, X chromosome transcriptional activation, and GLD-1 down-regulation.

The translation of the *rme-2* mRNA is delayed relative to its transcription by the binding of GLD-1 to its 3′ UTR [4]. GLD-1 levels fall precipitously at the end of pachytene, permitting translation of *rme-2* and other pro-oogenesis factors [4]. We examined RME-2 protein expression in *mpk-1* mutants and found that it does not accumulate to detectable levels (Figure 7B). Thus, in *mpk-1* mutants, the *rme-2* transcript is present but not translated, suggesting that loss of *mpk-1* blocks meiotic progression at a point at which GLD-1 is still present to prevent translation of target mRNAs. This observation is consistent with the finding that GLD-1 is not down-regulated in a *lin-45* (Raf) mutant, which shows a Pex phenotype similar to *mpk-1* mutants [32]. Combined, our analyses suggest that MPK-1 signaling occurs subsequent to EFL-1/DPL-1 activity but prior to GLD-1 down-regulation (Figure 8).

## Discussion

The identification of downstream effectors of signaling pathways in vivo will increase our understanding of how these commonly used pathways can produce the specific, desired outcome during development and organ function. Here, we have identified genes expressed subsequent to MPK-1 signaling in the germ line of *C. elegans,* using a combination of expression profiling, in situ hybridization, and mutant analysis. We demonstrated that one gene, which we named *pzf-1,* has MPK-1 signaling-dependent expression and potentially acts downstream of MPK-1 to promote proper segregation of chromosomes during meiosis. The identification of MPK-1 signaling-responsive genes also provided a means to examine the temporal relationships between MPK-1 signaling and other events in the pachytene stage, including EFL-1/DPL-1-targeted gene expression and X chromosome gene expression.

### Discovery of MPK-1 Signaling Effectors in the Germ Line

The *mpk-1(ga111)* mutant phenotype has three features that make it particularly suitable for identification of MPK-1 signaling-responsive genes in the germ line: tissue specificity, temperature sensitivity, and, as we show here, reversibility. The *ga111* lesion is a point mutation near the MEK binding domain and therefore most likely impairs the ability of MEK-2 to activate MPK-1 [9]. Presumably, the germ line is most sensitive to weak loss of MPK-1 activity, which is why *mpk-1(ga111)* does not result in a phenotype in other tissues known to require MPK-1. The reversibility of the Pex phenotype after shift of adults to the permissive temperature is consistent with a requirement for MPK-1 in an ongoing process, namely, meiotic progression.

The recovery of detectable MAP kinase activation and resumption of meiotic progression takes several hours after the shift to permissive temperature. The slow recovery of meiotic progression is probably due to a need to replace inactive MPK-1 with active MPK-1, as well as the length of time required for a germ cell to successfully execute the molecular events necessary to progress from pachytene through oogenesis. In wild-type, the time between pachytene exit and fertilization of the resulting oocyte is approximately 4–6 h at 25 °C [2]. The 9-h interval between shift to permissive temperature and visible changes in chromatin morphology in *mpk-1* mutants fall within this window, given that the rate of development at 15 °C is approximately twice as slow as at 25 °C [33]. Additionally, at the restrictive temperature, *mpk-1* germ cells stalled in late pachytene appear partially degenerated and might be incapable of meiotic progression upon shift to the permissive temperature, so that only cells in early pachytene can progress once MPK-1 signaling resumes. The oocytes produced after 15 h at the permissive temperature are functional, as they can be fertilized and generate live progeny. Thus, the *mpk-1(ga111)* mutant provided a valid system to investigate gene expression changes during meiotic progression, and most MPK-1 signaling-responsive genes identified using our experimental design are likely to be expressed during normal oogenesis.

Consistent with this expectation, we found that most MPK-1 signaling-dependent genes have germ-line-enriched expression based on an independent microarray dataset [19]. The strong enrichment for genes expressed in the germ line is probably due to the germ-line-specific phenotype of the *mpk-1(ga111)* allele and to our experimental design, which required that expression of these genes had to follow both loss and resumption of MPK-1 activity. In situ hybridization confirmed MPK-1 signaling-dependent expression in the germ line for seven out of eight genes tested—3/3 candidates on an autosome and 3/4 on the X. However, because microarray and in situ analyses measure only steady state mRNA levels, we cannot distinguish between direct and indirect effects on gene expression. MPK-1 may phosphorylate transcription factors that directly affect gene expression. Alternatively, MPK-1 phosphorylation substrates might promote meiotic progression through other mechanisms, with the gene expression changes we see as a secondary consequence. Until the phosphorylation substrates of MPK-1 are identified, we will be unable to test for direct regulation of target genes.

In general, downstream effectors of signaling pathways have not been identified by genetic screens, perhaps because signaling pathways target multiple, redundant effectors. In the germ line, MPK-1 may promote several aspects of meiotic progression through branching of the pathway to multiple downstream effectors. Any single MPK-1 signaling-dependent gene product may make only a small contribution to a process and result in a phenotype only in a sensitized background, as we found with the deletion of *pzf-1*. *pzf-1* deletion mutants exhibit no phenotype on their own but in combination with the *mpk-1(ga111)* mutation display a decreased brood size as well as increased embryonic lethality and incidence of male progeny, suggestive of defects in chromosome segregation. Other genes (perhaps other MPK-1 signaling-dependent genes) must contribute to the same process*,* as *pzf-1* is not absolutely required for meiotic progression (Figure 8A). The MPK-1 signaling-dependent genes identified in this study do not obviously implicate any one process in meiotic progression but are members of a variety of functional categories. While we have not yet examined the potential roles of most MPK-1 signaling-dependent genes, we suggest that others might function in meiotic progression, oogenesis, and/or embryogenesis.

### The Relationship between MPK-1 and EFL-1/DPL-1 in the Germ Line

Activated MAP kinase staining extends into the proximal germ line in *efl-1* mutant germ lines, indicating that EFL-1 negatively regulates MAP kinase activity at the end of the pachytene stage of meiosis I [2]. One prediction from this observation is that one or more EFL-1/DPL-1 target genes encode negative regulators of MPK-1 activity. Although *lip-1,* a known negative regulator of MPK-1, is not an EFL-1 target, other EFL-1 target genes could fulfill this role [3,29]. Another prediction from this observation is that genes regulated by MPK-1 would have elevated expression in *efl-1* mutants. Strikingly, of the eight genes commonly regulated by both pathways, six are down-regulated in *efl-1* mutants, opposite to this expectation. One possible explanation for this discrepancy is that increased MAP kinase activation in an *efl-1* mutant does not result in a significant increase in gene expression, either because MPK-1 activity is not sufficiently elevated or because ectopic MPK-1 cannot effectively regulate gene expression in the proximal germ line because transcription is shut off in maturing oocytes [6]. An alternate possibility is that we missed identifying many MPK-1 signaling-regulated genes, possibly because our microarray analysis was performed in whole animals, whereas the *efl-1* and *dpl-1* microarray analysis was performed on dissected gonads [3]. Additionally, the *mpk-1* allele we used for this analysis was not a null and might still regulate gene expression somewhat even at the restrictive temperature.

Conversely, the expression of EFL-1/DPL-1 target genes appears unaffected in *mpk-1* mutants as well. This observation is surprising because many EFL-1/DPL-1 target genes are known to play roles in oogenesis, and one might expect that their expression would be decreased in *mpk-1* mutants, which fail to make oocytes. In particular, we found that at least two EFL-1/DPL-1 target genes, *rme-2* and *cpg-3,* still have high expression levels in the proximal germ line of *mpk-1(ga111)* mutants. These data suggest that even though certain components of oocyte development are present in *mpk-1(ga111)* mutants, they are incapable of progressing beyond the pachytene stage. Thus, our comparisons of EFL-1/DPL-1 target genes and MPK-1 signaling-responsive genes demonstrate that these pathways influence the expression of almost completely different sets of genes. The overlap of only a few genes between the two suggests that they control distinct aspects of germ line development through different mechanisms.

### Temporal Relationship between MPK-1 Signaling and Other Events in Pachytene

Our identification of genes that depend on MAP kinase signaling in the germ line permitted us to analyze our results in the context of previously published data to make conclusions about the order of MAP kinase signaling relative to other events occurring in the pachytene stage of meiosis I. We found that the expression of MPK-1 signaling-responsive genes is first detectable in late pachytene, more proximal than expression of EFL-1/DPL-1 target genes (e.g., compare Figure 3 with *rme-2* in Figure 7). Additionally, *rme-2* and *cpg-3* mRNA is still present in *mpk-1(ga111)* mutants, although we cannot rule out that a stronger loss-of-function allele of *mpk-1* might reduce their expression. These data suggest that EFL-1/DPL-1 regulation occurs prior to MPK-1 signaling.

Several EFL-1/DPL-1 target genes, including *rme-2,* require down-regulation of the translation inhibitor GLD-1 in late pachytene prior to their translation [4]. Strikingly, we found that while *rme-2* mRNA is present in *mpk-1(ga111)* mutant gonads, the RME-2 protein was not present. This observation is consistent with previous data suggesting that down-regulation of GLD-1, and hence translation of RME-2, does not occur in *lin-45* mutants [32]. Therefore MPK-1 signaling likely occurs prior to GLD-1 down-regulation.

Our work also provides insight into the temporal relationship between pachytene exit and X chromosome activation. Although microarray analysis found only a subset of X chromosome genes as MPK-1 signaling-dependent genes, further examination by in situ hybridization showed that most X-linked, germ-line-expressed genes depend on MPK-1 for expression. This result suggests that MPK-1 signaling must occur in order for the X chromosomes to gain full transcriptional competence. Another event in late pachytene, physiological apoptosis of germ cells, has also been placed downstream of MPK-1 signaling [5]. MPK-1 could promote GLD-1 down-regulation, X chromosome reactivation, or apoptosis directly by regulating genes that control these events or indirectly by promoting meiotic progression to a point that allows temporally regulated events to occur.

### Conclusions

In summary, our analysis has further clarified the relationships between regulatory pathways that control meiotic progression and oogenesis in *C. elegans.* Comparison of the gene expression outputs of two regulatory pathways, such as the EFL-1/DPL-1 and MPK-1 pathways, contributes to our understanding of the functional relationships between these pathways. Additionally, further study of the MPK-1 signaling-dependent genes identified here will reveal more about the molecular mechanisms by which MPK-1 signaling influences meiotic progression. Determination of the contributions of multiple downstream effectors will aid in the identification of those with redundant functions. These studies will shed light on how commonly used signaling pathways mediate tissue-specific cell fates.

## Materials and Methods

### Strains and maintenance.

Nematode strain maintenance was as described in Brenner [34]. The following variants were used: N2 (wild-type), *dpl-1(n3316) unc-4(e120)* II [35], *unc-79(e1068) mpk-1(ga111)* III [9], *fem-1(hc17)* IV [15], *lip-1(zh15)* IV [29]*,* and *let-60(ga255)* IV (equivalent to *let-60(ga45)*) [28]. Strains containing *fem-1(hc17)* were maintained at 15 °C. Other strains were maintained at 20 °C. For *mpk-1(ga111)* microarray analysis and in situ hybridization, 26 °C was used as the restrictive temperature to ensure complete penetrance of the Pex phenotype. For analysis of the genetic interaction between *mpk-1* and *pzf-1,* 25 °C was used as the restrictive temperature. All strains with *mpk-1(ga111)* also carry *unc-79(e1068).*

### Immunofluorescence.

Antibody staining was performed as described in Kelly et al. [6]. Briefly, gonads were dissected, fixed in 3% paraformaldehyde, mounted on a slide, and incubated for 5 min in a humid chamber. Slides were frozen in liquid nitrogen and the cover slip cracked off before storing in methanol at −20 °C. The slides were washed with PBST (1× PBS with 0.1% Tween20), blocked in PBST with 0.5 mg/ml BSA, and then incubated overnight with α-diphosphorylated MAPK (1:100; Sigma, St. Louis, Missouri, United States) or α-RME-2 (1:100) [21]. Slides were then washed and incubated at room temperature for 2–3 h with a fluorescent secondary antibody (1:500, Molecular Probes, Carlsbad, California, United States). Slides were then stained with DAPI, washed in PBST, mounted in mounting medium with DABCO (Sigma), and viewed using a Zeiss (Oberkochen, Germany) Axioplan 2 imaging epifluorescence microscope.

### Microarray analysis.

Control *(unc-79(e1068);fem-1(hc17))* and mutant *(unc-79(e1068)mpk-1(ga111);fem-1(hc17))* worms were staged by bleaching gravid adults to collect eggs, which were then hatched in S-basal solution in the absence of food. The starved L1 larvae were plated with food (bacteria strain OP50) at 26 °C until the young adult stage, about 47 h. Worms were washed off and rinsed two times with M9 buffer. For each sample, total RNA from ~100 adult worms (4–5 μg) was isolated using Trizol (Invitrogen, Carlsbad, California, United States) and amplified with T7 RNA Polymerase using one round of linear amplification [36]. Three independent time courses were collected.

Fluorescence-labeled cDNA probe for DNA microarray hybridization was prepared from 4.5 μg of amplified RNA as described in DeRisi et al. [37]. A mixture of control RNA from the 0 and 15 h time points was used as a reference. Reference cDNA was labeled with Cy5 and compared to *mpk-1* cDNA labeled with Cy3. C. elegans whole-genome microarrays were used for hybridization as described in Jiang et al. [17]. Each slide was scanned using an Axon scanner (Molecular Devices, Sunnyvale, California, United States), and the expression levels for each gene in each channel were collected using GenePix 3.0 software. Cy5/Cy3 ratios were calculated and normalized by setting the overall median of ratios to one.

To determine which genes changed in response to MPK-1 signaling, we calculated the mean log ratio of 0-h control/reference compared to 0-h *mpk-1*/reference, and used a two-tailed, unpaired Student's *t*-test, without correction for multiple testing, to identify genes with statistically significant changes. We chose genes with a log_2_ ratio > 0.7 (~1.6-fold) and *p* < 0.05 as MPK-1 signaling-responsive. Using hierarchical clustering and the Cluster and Treeview programs [18], these genes were then grouped by the log_2_ ratio of *mpk-1*/reference from the 0 through 15-h time points. The data were adjusted by normalizing each gene before undergoing average linkage clustering. The 15-h time point was not weighted in the clustering analysis because it showed a decrease in the log_2_ ratio of *mpk-1*/reference for most genes. We speculate that this decrease occurs because the absence of sperm results in a halt in oogenesis and meiotic progression. The clustering analysis defined three major groups of genes, each with a correlation of expression greater than 0.95.

X-linked genes with oogenic germ-line-enriched expression that displayed mild response to MPK-1 signaling were identified as those having a 1.4-fold or greater increase in expression at one or more time points after the shift to permissive temperature relative to the 0-h time point.

### In situ hybridization.

In situ hybridization was performed essentially as described in Jones et al. [38]. Probes were prepared from full-length or partial cDNAs cloned in pCRII or pCR2.1 vector (Invitrogen). cDNA fragments used for each probe were as follows: F16H11.3, nucleotides (nt) 12–1300; K02B9.1, nt 4–2585; C49F5.3, nt 55–770; F35C8.7, nt 1–2227; C46C11.1, nt 1190–2810; C17H11.2, nt 1–1822; F16B12.6, nt 1883–3190; C04A11.3, nt 1628–2830; *pzf-1,* nt 4–1584; *hlh-2,* nt 1–1198; *sea-1/tbx-18,* nt 1–1131, and *rme-2,* nt 1–1029, where numbering begins at the ATG in the predicted spliced cDNA. cDNAs were amplified from ~2 ng of plasmid using gene-specific primer pairs. *cpg-3* was amplified from a plasmid containing nt −108 to 1048 from the genomic locus of R06C7.4. Purified PCR product (800 ng) was used with a single gene-specific primer for repeated primer extension with digoxigenin-11-dUTP to label either a sense or AS single-stranded probe. Probes were diluted 1:2 in hybridization buffer then applied to gonads and hybridized at 48 °C for 24–30 h. Following hybridization and washing, the gonads were incubated with alkaline-phosphatase-conjugated anti-Dig (Fab2 fragment) (Roche, Indianapolis, Indiana, United States) at 4 °C overnight. Gonads were stained with BCIP/NBT tablets (Sigma or Roche), mounted, and viewed using a Zeiss Axioplan 2 imaging epifluorescence microscope.

### Deletion mutant identification.

Deletion alleles for selected genes were obtained by PCR screening a library of mutagenized worms for deletions in the desired locus. Deletion library construction and screening were performed as described in Ahringer [39]. Breakpoints of deletions isolated are as follows: *pzf-1(vr3),* cacagattccgcaaagtaac/gggtttcgaaaatcgtattt; *sea-1/tbx-18(vr4),* agaggtaggaagatagacacgaa/ggagtcttctttctgattgcaat; and T08D10.2*(vr12),* aattgtttaaaattccatta/acaagaatctaaaacaaaat. After identifying candidate deletion mutations, frozen worms from corresponding wells were recovered and homozygous mutants isolated. Prior to phenotypic and genetic analysis, *vr3* and *vr4* were backcrossed six times to remove potential additional mutations. The ovulation rate was determined by counting the number of oocytes and/or embryos laid on a seeded plate during a 1.5–2 h time period. This number was used to calculate ovulations/gonad arm/hour.

## Supporting Information

Table S1 MAP Kinase Signaling-Responsive Genes (18 KB PDF)

### Accession Numbers

All microarray analysis data are available in Gene Expression Omnibus (GEO) (http://www.ncbi.nlm.nih.gov/geo) under accession number GSE5069.

## Abbreviations

AS: antisense
nt: nucleotides
Pex: pachytene exit defect
*pzf-1*: *paired zinc finger-1*
