# Supplementary material for: Expression Profiling of MAP Kinase–Mediated Meiotic Progression in Caenorhabditis elegans
Source: PLoS Genet. 2006 Nov 10;2(11):e174. doi: 10.1371/journal.pgen.0020174 (PMC1635537; doi:10.1371/journal.pgen.0020174)
Supplement: Supplementary file 1 [file pgen.0020174.st001.pdf]

Supplemental Table 1. MPK-1 Signaling-Dependent Genes

MPK-1 Signaling-Responsive: Strong

			Regulation (log2)									Expression Pattern		Oogenic
			control/ mpk-1	reference/mpk-1										Germline
Wormbase ID	Name	Chr.	*0hr*	*0hr*	*3hr*	*6hr*	*9hr*	*12hr*	*15hr*	Function	RNAi	In situ pattern	EST	Enriched
C14A11.3	*tag-150*	X	0.72	0.71	0.68	0.36	0.02	0.18	0.73	Signaling	WT	no image	yk601b9	yes
C17E7.4		V	1.04	1.08	0.76	0.49	0.09	-0.24	0.92	Unknown	Emb/WT	proximal germline, embryo	yk502d2	yes
C55B6.1		X	0.89	1.03	0.74	0.41	-0.14	-0.33	0.47	Metabolism	WT	no staining	yk735b7	yes
F02H6.3		IV	0.80	0.84	0.64	0.46	-0.01	-0.52	0.24	Unknown	WT	proximal germline	yk258a8	yes
F08F3.6		V	1.00	1.24	1.01	0.61	-0.07	-0.16	0.69	Proteolysis	WT	proximal germline, embryo	yk267c7	yes
F14D7.2		V	1.03	1.55	0.96	0.53	0.15	-0.24	0.81	Unknown	Rup/WT	proximal germline	yk502c10	yes
F14H3.4		V	0.81	1.19	0.96	0.57	-0.06	-0.20	0.70	Proteolysis	WT	no image	no ESTs	yes
F14H3.5		V	0.72	1.11	0.80	0.39	0.07	0.09	0.83	Unknown	WT	no image	no ESTs	yes
F26G5.1*		V	1.07	1.77	1.22	0.76	0.28	0.27	0.72	Unknown	Emb/WT	medial germline, embryo	yk166d9	yes
F35C8.7*		X	1.27	1.35	1.20	0.65	0.15	-0.14	0.65	Membrane	WT	proximal germline, embryo	yk90d4	yes
F52D2.2	*rgs-8.1*	X	1.05	1.07	0.93	0.54	-0.01	-0.17	0.70	Signaling	WT	no image	no ESTs	yes
K02B9.1		X	1.11	1.25	0.76	0.32	0.02	-0.16	0.59	Unknown	WT	proximal germline	yk338b7	yes
K02B9.2		X	0.90	1.08	0.74	0.43	0.11	0.16	0.45	Unknown	WT	proximal germline	yk472c1	yes
K04C1.5*		X	1.02	1.31	0.91	0.45	-0.04	0.05	0.78	Signaling	Emb/WT	no image	no ESTs	yes
T02G6.5		I	1.14	1.55	1.20	0.78	0.07	0.14	0.70	Proteolysis	WT	broad germline, larval mus	yk497a11	yes
T05G11.1	*pzf-1*	V	1.36	1.62	1.16	0.77	0.18	-0.03	0.65	Transcription	WT	no image	no ESTs	yes
T08D10.1	*tag-295*	X	0.74	1.04	0.76	0.52	0.22	-0.01	0.66	Transcription	WT	broad germline embryo	yk468e11	yes
T23C6.3		X	1.03	1.01	0.87	0.41	0.05	-0.06	0.21	Unknown	WT	intestine	yk634e1	no
ZC53.7	*rgs-9*	X	0.72	0.58	0.50	0.28	-0.19	-0.16	0.46	Signaling	Emb/WT	no staining	yk374h2	yes
ZK177.1		II	0.82	1.21	0.83	0.41	0.02	-0.07	0.72	Unknown	WT	no staining	yk603b8	yes
ZK637.11	*cdc-25.3*	III	1.29	1.23	1.01	0.46	0.23	0.11	0.74	Signaling	WT	proximal germline, embryo	yk631b11	yes

MPK-1 Signaling-Responsive: Medium

			Regulation (log2)									Expression Pattern		Oogenic
			control/ mpk-1	reference/mpk-1										Germline-
Wormbase ID	Name	Chr.	*0hr*	*0hr*	*3hr*	*6hr*	*9hr*	*12hr*	*15hr*	Function	RNAi	In situ pattern	EST	Enriched
B0410.2	*vang-1*	X	1.15	1.68	1.27	1.11	0.74	0.49	1.33	Membrane	Emb/WT	broad germline, embryo	yk594e5	no
C29F7.4	*fkh-3*	X	1.44	1.56	1.47	1.12	0.43	0.11	0.78	Transcription	WT	proximal germline, embryo	yk499c1	yes
C35E7.1		I	1.96	2.24	1.84	1.53	1.00	0.69	1.35	Unknown	WT	embryo	yk601c12	yes
C49F5.3		X	0.96	1.20	0.94	0.68	0.48	0.20	0.76	Unknown	WT	no image	no ESTs	yes
C54H2.3	*tag-294*	X	0.96	1.32	1.10	0.76	0.35	0.11	0.81	Unknown	WT	proximal germline	yk593h1	no
F16H11.3*		X	0.81	1.15	0.78	0.55	0.33	0.14	0.84	Metabolism	WT	no image	no ESTs	yes
F19B10.10		II	0.74	1.29	0.99	0.87	0.37	0.40	0.89	Unknown	WT	proximal germline	yk563e3	no
F19B10.9	*sea-1/tbx*	II	1.13	1.45	1.10	1.06	0.52	0.39	0.97	Transcription	WT	no staining	yk245a1	yes
F45C12.15		II	0.88	1.25	1.03	0.78	0.26	0.24	0.89	Transcription	WT	proximal germline, embryo	yk469g5	yes
F54D5.5		II	0.86	1.48	1.16	0.80	0.32	0.51	1.09	Unknown	Bmd/Unc	proximal germline, embryo	yk389a6	yes
F56B6.2	*rgs-7*	X	1.01	1.33	0.86	0.77	0.34	0.33	0.83	Signaling	WT	proximal germline	yk46a8	yes

Supplemental Table 1. MPK-1 Signaling-Dependent Genes

F57C7.1a		X	0.86	1.34	1.17	0.89	0.47	0.33	0.91	Transcription	WT	proximal germline	yk132f4	yes
F59A3.9		I	1.13	1.57	1.28	0.90	0.48	0.07	0.99	RNA	WT	no staining	yk331h9	no
T03G11.6		X	0.74	1.17	0.94	0.94	0.34	0.35	0.96	Unknown	WT	no image	yk1520c09	yes
T05H10.4		II	1.31	1.84	1.47	0.88	0.30	0.50	1.00	Unknown	WT	proximal germline	yk114d3	yes
T08D10.2		X	0.93	1.22	1.04	0.66	0.34	0.23	0.56	Transcription	WT	proximal germline, embryo	yk167h2	yes
Y11D7A.13		IV	1.28	1.63	1.43	0.81	0.33	0.22	0.92	Unknown	WT	no image	no ESTs	no
Y24F12A.4		IV	0.92	1.17	0.80	0.91	0.24	0.25	1.08	Unknown	WT	no image	no ESTs	no
Y57G11C.25		IV	0.82	0.94	0.65	0.56	0.27	0.11	0.82	RNA	WT	no image	no ESTs	yes
Y9D1A.1		II	0.99	1.02	0.70	0.76	0.23	0.20	0.77	Unknown	No data	proximal germline nuclei	yk610e5	no
ZC376.4		V	0.93	0.97	0.81	0.61	0.43	0.26	1.00	Transcription	WT	no image	no ESTs	no

MPK-1 Signaling-Responsive: Weak

			Regulation (log2)											
			control/ mpk-1	*reference/mpk-1*								Expression Pattern		Oogenic Germline Enriched
Wormbase ID	Name	Chr.	*0hr*	*0hr*	*3hr*	*6hr*	*9hr*	*12hr*	*15hr*	Function	RNAi	In situ pattern	EST	
B0272.2		X	0.85	1.09	0.72	0.61	0.34	0.54	1.06	Folding/Sortin	Emb/WT	no image	no ESTs	yes
C07D8.6*		X	0.85	0.82	0.72	0.55	0.26	0.35	0.90	Metabolism	WT	proximal germline, embryo	yk609e5	yes
C23H3.1	*egl-26*	II	0.82	1.35	1.06	0.90	0.45	0.53	1.17	Unknown	Lva/Emb	proximal germline	yk639g4	yes
C31G12.1		V	0.89	1.70	1.05	0.99	0.90	0.61	1.05	Unknown	WT	no image	no ESTs	yes
C32F10.6	*nhr-2*	I	1.08	1.95	1.64	1.45	0.70	1.13	1.71	Transcription	Emb/Lva	no staining	yk487a11	no
C35B8.3		X	0.76	0.95	0.47	0.45	0.27	0.49	0.54	Unknown	WT	broad germline, embryo	yk614e3	no
C52D10.6	*skr-12*	IV	1.47	2.17	1.98	1.68	1.46	1.39	2.43	Proteolysis	Ste/WT	no image	yk344e2	no
C52D10.9	*skr-8*	IV	1.07	1.27	1.47	1.38	0.92	0.71	1.98	Proteolysis	Bmd/Ste	embryo	yk666c7	yes
D1009.2	*cyn-8*	X	1.07	1.56	1.07	1.13	0.75	0.86	0.98	Folding/Sortin	WT	proximal germline, embryo	yk601g8	no
E01G4.5		II	0.81	0.98	0.96	0.89	0.35	0.45	1.07	Proteolysis	WT	faint germline, embryo	yk388g3	yes
F13A7.9	*skr-11*	V	0.85	1.64	1.08	0.83	0.57	0.96	1.71	Proteolysis	WT	no image	no ESTs	yes
F22B8.7		V	1.24	1.98	0.97	0.82	0.54	0.53	1.21	Unknown	WT	intestine, spermatheaca	yk438f6	no
F22F4.2	*inx-3*	X	1.33	2.32	2.07	1.83	1.25	1.20	1.63	Structure	Gro/Unc	broad germline, embryo	yk563a4	yes
F25H9.4	*bath-39*	V	0.73	1.20	0.79	0.82	0.47	0.45	1.15	Proteolysis	WT	no image	no ESTs	no
F29G9.1		V	1.03	1.68	1.06	1.12	0.60	0.77	1.38	Unknown	WT	no image	no ESTs	yes
F35E12.3*		V	0.75	1.18	1.02	0.69	0.48	0.69	1.18	Proteolysis	WT	no image	no ESTs	yes
F44A6.2	*sex-1*	X	1.36	2.17	2.07	1.69	1.32	0.76	1.77	Transcription	Bmd/Dpy	broad germline, embryo	yk593a10	no
F44F1.7	*vet-6*	I	1.25	1.80	1.50	1.59	1.00	1.50	2.54	Structure	WT	embryo	yk531c12	no
F45C12.7		II	0.89	1.36	1.37	1.16	0.81	0.79	1.45	Proteolysis	Sle/WT	faint staining	yk594e6	yes
F46A9.6	*mec-8*	I	0.72	1.25	0.88	0.77	0.62	0.65	1.30	RNA	WT	embryo, larvae, adult	yk607c4	no
F56D5.5		IV	1.04	1.59	1.43	1.39	0.70	0.71	1.33	Structure	WT	broad germline, vulva, emb	yk534d1	yes
F56G4.2**	*pes-2*	I	1.67	2.46	2.76	2.78	1.74	1.94	4.06	Proteolysis	WT	no image	no ESTs	yes
F56G4.3**		I	1.90	2.68	2.74	2.88	1.81	2.15	3.95	Proteolysis	WT	embryo	yk522g11	yes
K09H9.3	*col-49*	I	0.73	-0.15	-0.10	0.05	0.09	-0.12	0.69	Structure	Dpy/WT	no image	no ESTs	no
M01E5.6		I	1.46	2.00	1.84	1.84	1.03	1.62	2.29	Transcription	WT	embryo	yk593g11	no
M05B5.5	*hlh-2*	I	0.86	1.80	1.39	1.35	0.83	0.82	1.72	Transcription	Emb/Stp	no staining	yk492c11	yes
R05H11.1		III	0.80	1.06	0.86	0.81	0.53	0.58	1.30	Proteolysis	WT	no staining	yk857h09	yes

Supplemental Table 1. MPK-1 Signaling-Dependent Genes

R90.1		V	0.70	1.09	0.71	0.71	0.38	0.44	1.05	Signaling	Lva/Unc	no staining	yk607h3	no
T09B4.5		I	1.14	2.77	2.40	2.11	1.62	1.74	2.61	Transcription	WT	embryo	yk606a4	no
T13F2.8	*cav-1*	IV	1.47	3.25	2.44	2.43	1.58	1.71	2.66	Signaling	WT	embryo	yk112g9	yes
T17A3.3	*fbxb-81*	III	0.81	1.09	1.04	1.09	0.76	0.95	1.83	Proteolysis	WT	no image	no ESTs	no
T17A3.4	*fbxb-82*	III	0.74	1.00	0.93	1.03	0.73	0.68	1.70	Proteolysis	WT	no image	no ESTs	yes
T22B7.4		X	0.86	1.55	1.12	1.04	0.70	0.83	1.39	Transcription	WT	faint staining	yk590c10	no
T25E12.6		V	1.06	1.82	1.34	1.28	0.97	1.12	2.03	Unknown	WT	broad germline	yk572h8	no
T27D12.4	*pes-5*	II	0.79	1.32	1.03	0.96	0.58	0.65	1.29	Unknown	WT	medial germline	yk187h1	yes
W01A11.5	*cpt-6*	V	0.77	1.53	1.35	1.28	0.72	0.73	1.29	Metabolism	WT	medial germline	yk499f4	no
Y47D7A.1	*skr-7*	V	1.70	2.24	2.32	2.01	1.29	1.32	1.99	Proteolysis	Bmd/Em	distal germline, embryo	yk537b10	yes

MPK-1 Signaling-Dependent: Not Responsive

			Regulation (log2)									Expression Pattern		Oogenic
			control/ mpk-1	*reference/mpk-1*										Germline
Wormbase ID	Name	Chr.	*0hr*	*0hr*	*3hr*	*6hr*	*9hr*	*12hr*	*15hr*	Function	RNAi	In situ pattern	EST	Enriched
C50B6.4	*col-161*	V	0.83	0.23	0.18	0.17	0.09	0.40	1.04	Structure	WT	somatic gonad	yk59c1	no
F35E12.5		V	1.79	1.26	1.44	1.65	1.63	0.98	2.67	Membrane	WT	no image	no ESTs	no
F35E12.6		V	0.81	0.47	0.68	0.48	0.25	0.30	1.15	Membrane	WT	no image	no ESTs	no
F43G6.5		II	0.81	1.38	0.99	0.79	0.99	1.18	1.83	RNA	WT	embryo	yk484e9	no
F57B1.4	*col-160*	V	0.70	0.12	0.18	0.24	0.11	0.53	1.21	Structure	WT	no image	yk102e6	no
K09E9.3		X	0.75	0.85	0.84	0.60	0.35	0.38	0.68	Structure	WT	embryo	yk536a2	no
M60.2		X	1.04	1.92	0.62	-0.23	-0.22	-0.14	0.87	Unknown	WT	intestine	yk177g5	no
T27A8.2		X	0.77	0.45	0.37	0.19	-0.14	0.19	0.72	Transcription	WT	no image	no ESTs	yes
Y2H9A.3	*col-162*	V	0.82	0.08	0.03	0.11	-0.02	0.26	1.09	Structure	WT	somatic gonad	yk59c1	no
Y46G5A.27	*pes-10*	II	0.85	0.94	0.75	0.67	0.45	0.88	1.35	Proteolysis	WT	larval soma, embryo	yk497a10	yes
ZK899.6		X	0.72	0.77	0.71	0.59	0.41	0.84	1.56	Unknown	WT	no image	no ESTs	no

Upregulated in *mpk-1(ga111)* mutants

			Regulation (log2)									Expression Pattern		Oogenic
			control/ mpk-1	*reference/mpk-1*										Germline
Wormbase ID	Name	Chr.	*0hr*	*0hr*	*3hr*	*6hr*	*9hr*	*12hr*	*15hr*	Function	RNAi	In situ pattern	EST	Enriched
B0511.11		I	-1.31	-1.08	-0.84	-0.62	-0.44	-0.48	0.20	Unknown	WT	somatic gonad	yk39e9	no
C34G6.1		I	-0.74	-0.70	-0.89	-0.38	-0.29	-0.09	0.07	Unknown	Ste	no image	yk346c10	yes
C05C10.4		II	-0.84	-0.43	-0.23	-0.25	-0.50	-0.18	0.75	Metabolism	WT	posterior all stages	yk128f9	no
F35C11.5		II	-0.77	-0.62	-0.40	-0.31	-0.28	-0.12	0.76	Unknown	WT	broad germline, embryo	yk78f12	yes
F54B3.1		II	-0.79	-0.78	-0.42	-0.24	-0.37	-0.79	0.49	Unknown	Emb/WT	faint germline	yk134e3	no
F59B2.2		III	-0.78	-0.28	-0.22	-0.24	0.09	0.26	0.87	Membrane	WT	broad germline, embryo	yk484a7	yes
K08H10.1*	*lea-1*	V	-1.33	-0.95	-0.97	-0.69	-0.81	-0.63	0.16	Neuro	WT	broad germline	yk43h9	no

*downregulated in *efl-1* and *dpl-1* mutants
**upregulated in *efl-1* and *dpl-1* mutants
